# Free Transplantation of a Tissue Engineered Bone Graft into an Irradiated, Critical-Size Femoral Defect in Rats

**DOI:** 10.3390/cells10092256

**Published:** 2021-08-31

**Authors:** Ulrike Rottensteiner-Brandl, Ulf Bertram, Lara F. Lingens, Katrin Köhn, Luitpold Distel, Tobias Fey, Carolin Körner, Raymund E. Horch, Andreas Arkudas

**Affiliations:** 1 Department of Plastic and Hand Surgery, University Hospital Erlangen, Friedrich-Alexander University Erlangen-Nürnberg (FAU), 91054 Erlangen, Germany; ulrike.rottensteiner@fau.de (U.R.-B.); ubertram@ukaachen.de (U.B.); llingens@ukaachen.de (L.F.L.); koehnkatrin@googlemail.com (K.K.); Raymund.Horch@uk-erlangen.de (R.E.H.); 2 Emil-Fischer Zentrum, Institute of Biochemistry, Friedrich-Alexander University Erlangen-Nürnberg (FAU), 91054 Erlangen, Germany; 3 Department of Neurosurgery, RWTH Aachen University, 52074 Aachen, Germany; 4 Hand Surgery—Burn Center, Department of Plastic Surgery, University Hospital RWTH Aachen, 52074 Aachen, Germany; 5 Department of Radiation Oncology, University Hospital Erlangen, Friedrich-Alexander University Erlangen-Nürnberg (FAU), 91054 Erlangen, Germany; Luitpold.Distel@uk-erlangen.de; 6 Department of Materials Science and Engineering, Institute of Glass and Ceramics, Friedrich-Alexander University Erlangen-Nürnberg (FAU), 91054 Erlangen, Germany; tobias.fey@fau.de; 7 Frontier Research Institute for Materials Science, Nagoya Institute of Technology, Nagoya 466-8555, Japan; 8 Department of Materials Science and Engineering, Institute of Science and Technology of Metals, Friedrich-Alexander University Erlangen-Nürnberg (FAU), 91054 Erlangen, Germany; carolin.koerner@ww.uni-erlangen.de

**Keywords:** bone tissue engineering, critical size defect, irradiation, mesenchymal stem cells, hydroxyapatite

## Abstract

Healing of large bone defects remains a challenge in reconstructive surgery, especially with impaired healing potential due to severe trauma, infection or irradiation. In vivo studies are often performed in healthy animals, which might not accurately reflect the situation in clinical cases. In the present study, we successfully combined a critical-sized femoral defect model with an ionizing radiation protocol in rats. To support bone healing, tissue-engineered constructs were transferred into the defect after ectopic preossification and prevascularization. The combination of SiHA, MSCs and BMP-2 resulted in the significant ectopic formation of bone tissue, which can easily be transferred by means of our custom-made titanium chamber. Implanted osteogenic MSCs survived in vivo for a total of 18 weeks. The use of SiHA alone did not lead to bone formation after ectopic implantation. Analysis of gene expression showed early osteoblast differentiation and a hypoxic and inflammatory environment in implanted constructs. Irradiation led to impaired bone healing, decreased vascularization and lower short-term survival of implanted cells. We conclude that our model is highly valuable for the investigation of bone healing and tissue engineering in pre-damaged tissue and that healing of bone defects can be substantially supported by combining SiHA, MSCs and BMP-2.

## 1. Introduction

Large bone defects occur in several clinical situations, such as severe trauma, posttraumatic nonunion, tumor resection and osteomyelitis [1,2,3,4]. The current gold standard for treatment consists in filling the defect with autologous bone grafts [5], a method that carries two major drawbacks, namely the limited graft availability and the risk of donor site morbidity [6]. The use of allografts bypasses donor site morbidity if material from dead donors or from femoral heads at primary total hip replacements is used [5]. However, the transmission of diseases remains a matter of concern in allogenic transplantation [7]. The use of freeze-dried bone material allows for unlimited availability and sterilization by gamma irradiation, however carrying the downside of non-vital material and limited osteogenic properties [7]. All allogenic transplants share the risk of inducing an immune reaction in the host [6]. Considering the potential drawbacks of all different therapeutic options, further research is highly needed to develop new treatment modalities.

Over the past decades, the emerging field of regenerative medicine and tissue engineering has brought new perspectives for the treatment of large bone defects, combining anorganic or organic scaffolds, osteogenic cells and growth factors [5]. A tremendous number of different scaffold components and growth factors in tissue-engineered bone grafts have been investigated [6]. These synthetic substances are commonly combined with bone marrow-derived osteogenic cells or other cell types, usually after in vitro expansion [8]. Recently, unexpanded bone marrow mononuclear cells are being investigated for their usefulness in bone regeneration [9].

Tissue-engineered bone constructs necessarily need to be evaluated in animal models, as the interplay between the construct, surrounding bone tissue, vascularization and the immune system is a complex process that is only in part reflected in vitro. The vast majority of the published experimental in vivo studies use healthy, juvenile or young-adult animals as models [10]. Contrary to this, bone tissue surrounding clinically occurring bone defects is often damaged, i.e., by infection, irradiation or severe trauma. Therefore, results from healthy bone tissue models might not be readily applicable in clinical situations.

The goal of the current study was the implantation of an artificial, tissue-engineered bone substitute into a critical-size femoral defect (CSFD) in rats for evaluation of its therapeutic potential. In order to simulate pre-damaged bone tissue, a previously established irradiation model was applied. With this approach, results from studies in healthy animal bone tissue should be brought one step closer to its application in clinically occurring diseases.

## 2. Materials and Methods

### 2.1. Experimental Design

This research project was carried out as a prospective experimental study. Involved animals (rats) were divided into donor and acceptor individuals (see Table 1).

Each donor animal was used for subcutaneous prevascularization and preossification of one tissue-engineered construct that was later implanted into a critical size femoral defect in one corresponding acceptor animal. A combination of scaffold material (SiHA; silicon substituted hydroxyapatite, 0.8% Si; Actifuse, Baxter Healthcare GmbH, Vienna, Austria; granular size 2–5 mm), recombinant human bone morphogenetic protein 2 (rhBMP-2; Induct Os/Dibotermin alfa, Wyeth/Pfizer, Berlin, Germany; final concentration 100 μg/mL) and mesenchymal stem cells from syngenic donors after early osteogenic in vitro differentiation (3 × 10^6^ cells per construct) in fibrin glue (Tisseel Fibrin Sealant, Baxter Healthcare GmbH, Vienna, Austria; 10 mg/mL fibrinogen, 2 IE/mL thrombin) was used for osteogenesis in donor animals (groups C, C-20 Gy ionizing radiation (irrad), D and D-irrad). In groups B and B irrad, only scaffold in fibrin glue was used to evaluate osteogenic properties of the scaffold alone.

Acceptor animals were either irradiated 4 weeks prior to CSFD surgery (“irrad”) or left as unirradiated controls. During CSFD surgery, tissue-engineered constructs from donor animals were transferred to the acceptor animals (groups B/B irrad, C/C irrad, D/D irrad). An empty titanium chamber was used to bridge control defects, which served to prove the critical size of the bone defect (group A/A irrad).

### 2.2. Animals

A total number of 137 rats was used for surgical procedures in this study. Additionally, three animals were sacrificed for the isolation of mesenchymal stem cells from bone marrow. All experiments were performed using adult male Lewis rats, an inbred strain provided by Charles River Laboratories (Sulzfeld, Germany). Experimental handling was approved by the ethics committee of the government of Middle Franconia (Az 54-2532.1-9/13-2). Animals were kept under standardized conditions (20–22 °C, relative humidity 46–48%, 12 h light–dark cycle) with ad libitum access to water and food (Ssniff, Altromin, Lage, Germany). Animals were allowed to acclimatize for at least one week before starting the experiments.

### 2.3. Isolation and Culture of Mesenchymal Stem Cells

For isolation of mesenchymal stem cells, three animals were anesthetized by inhalation of isoflurane (Forene, Abbott, Wiesbaden, Germany) in pure oxygen. Both hindlimbs were removed under aseptic conditions, and the donor animals were anesthetized under deep anesthesia (T61, Intervet, Unterschleißheim, Germany). Femora and tibiae were aseptically removed from the surrounding soft tissue, and the bone marrow was flushed out using a syringe and needle using phosphate-buffered saline (PBS; Biochrom, Berlin, Germany) supplemented with 2% fetal calf serum (FCS Superior, Biochrom). The resulting cell suspension was centrifuged, resuspended in fresh growth medium (DMEM/Ham’s F-12, Biochrom, 1% L-glutamine, Gibco/Life Technologies, Darmstadt, Germany, 20% FCS Superior, Biochrom) and filtered through a 100 µm cell strainer. Mononuclear cells were isolated using density-gradient centrifugation (Histopaque^®^-1077, Sigma Aldrich, Seelze, Germany), and cells were plated at a density of 2 × 10^6^ mononuclear cells per well in a 24-well-plate. Non-adherent cells were removed after 36 h. Cells were passaged at a confluency of 80% using trypsin/EDTA (Biochrom). At the fourth passaging, cells were frozen until further use.

To confirm the mesenchymal stem cell phenotype of the cultured cells, the presence of MSC surface markers CD29, CD90, CD73 and CD54 and the absence (<5%) of leucocyte marker CD45 and endothelial cell marker CD31 was confirmed by flow cytometry (FACScalibur, BD Bioscience, Heidelberg, Germany; FlowJo Software, TreeStar, Ashland, OR, USA; antibodies and results of flow cytometry: please see Supplementary Table S1). Furthermore, the osteogenic differentiation potential was confirmed using a commercially alkaline phosphatase detection kit (Leucocyte Alkaline Phosphatase Kit, Sigma Aldrich, Seelze, Germany) after culturing in an osteogenic differentiation medium (growth medium supplemented with 25 μg/mL L-ascorbic acid, 10 mM glycerol 2 phosphate, 100 nM dexamethasone; all supplements Sigma Aldrich) (see Supplementary Figure S1).

Prior to in vivo implantation into donor animals, the cells were kept in an osteogenic differentiation medium for 5–6 days. The cells were labeled with red fluorescent dye for later detection on histological slides (DiI Cell Tracker, Life Technologies, Darmstadt, Germany).

### 2.4. Preparation of Donors

The subcutaneous implantation of chambers into donor animals was performed 6 weeks before CSFD surgery. For medication and anesthesia dosages, please see Appendix A.1.1.). A longitudinal incision was made in the left groin of donor animals, centered over the left femoral artery and vein. Subcutaneous tissue was bluntly separated, the femoral artery and vein were carefully separated from the underlying tissue, cauterized and dissected. This was done in order to avoid excessive bleeding from these large vessels after implantation of the titanium chamber.

**Figure 1:**
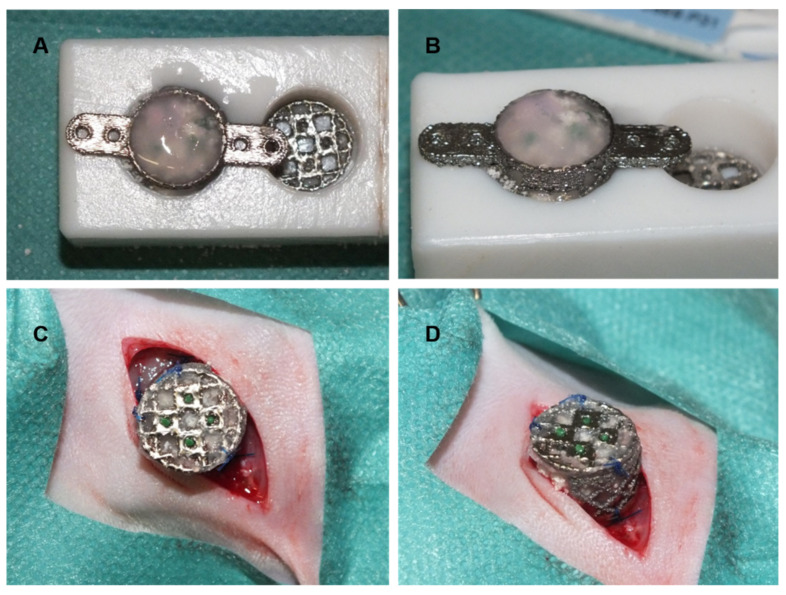
Implantation of chambers into donor animals. The chamber was filled with hydroxyapatite scaffold and fibrin sealant with or without rhBMP-2/mesenchymal stem cells after osteogenic differentiation (**A**,**B**). Afterward, the chamber was secured to the muscles in the medial femoral region (**C**,**D**).

A previously described custom-made titanium chamber was used (Supplementary Figure S2) [11]. The diameter of the chamber was 12 mm, the height was 10 mm, and the wings for later fixation to the femoral bone were 6 mm each, with two holes of 1.3 mm on each wing for the later placement of screws. The pore size of the cage forming the chambers’ wall was 2 mm to allow for the ingrowth of vessels from the surrounding tissue. Prior to implantation into the donor animals’ groin, the titanium chamber was filled with the scaffold material and fibrin glue with or without rhBMP-2 and mesenchymal stem cells (Figure 1A,B). The chamber was secured to the underlying muscle using non-resorbable suture material ((USP 4/0, polypropylene; Figure 1C,D), and the incision was closed after extensive flushing using resorbable suture material (USP 4/0, polyglactin 910).

### 2.5. Irradiation of Acceptor Animals

The irradiation procedure was carried out 4 weeks prior to CSFD surgery, based on the results of a previous study [12]. Briefly, acceptor animals were anesthetized (see Appendix A.1.2.) and the irradiation field was clipped and marked. The rats were placed in an IVC (individually ventilated cage) in ventral recumbency, with the left hindlimb extended laterally and kept parallel to the ground. The cage was closed in order to maintain SPF status (specific pathogen-free) throughout the irradiation procedure. Irradiation was carried out at the Department of Radiation Oncology (University Hospital Erlangen, Friedrich-Alexander-University Erlangen-Nurnberg) using an X-ray device (0.3 mm copper filtered, 120 kV, 24.2 mAs). A total ionizing radiation dose of 20 Gy was delivered in one session (1 Gy per minute) 4 weeks prior to CSFD surgery. At the time of CSFD surgery, the skin was intact in all animals.

### 2.6. CSFD Surgery

**Figure 2:**
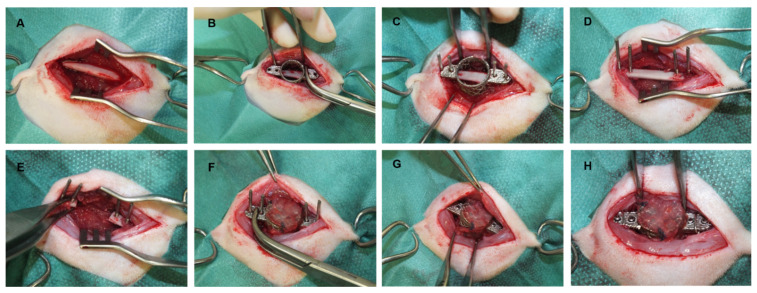
CSFD surgery. After exposure of the femur (**A**), Kirschner wire pins were positioned using an empty template chamber (**B**–**D**), and a 10 mm piece of the femoral shaft was removed (**E**). The prevascularized construct was transferred from the donor to the acceptor animal and secured with screws and a cerclage wire (**F**–**H**).

Four weeks after irradiation of the acceptor animals and six weeks after implantation of the titanium chamber into donor animals, both animals were anesthetized (see Appendix A.1.3.). The site of chamber implantation in donor animals and the lateral femoral area in acceptor animals were clipped and aseptically prepared. The CSFD was created as previously described [11]. Briefly, the skin over the lateral femur was incised, and subcutaneous tissue, muscular layers and periosteum were bluntly dissected to gain access to the femoral bone (Figure 2A). An empty titanium chamber was positioned over the femur and secured with surgical forceps (Figure 2B). The holes on the wedges of the chamber were used as a template to correctly determine the position of the screw holes. Using a microsurgical drill, four Kirschner wires (1.2 mm diameter) were positioned in the femoral shaft (Figure 2C). Then the template chamber was removed (Figure 2D), and a 10 mm long defect was created between the prepositioned Kirschner wires using a microsurgical bone saw (Figure 2E). The wound bed was carefully flushed to remove bone pieces and covered with a sterile, moist gauze. The implanted chamber was carefully removed from the donor animal, and donor animals were euthanized in deep anesthesia (T61, Intervet, Unterschleißheim, Germany). The prevascularized, preossified chamber was then positioned into the CSFD using the Kirschner wires as a guide (Figure 2F), and the wires were replaced by screws (1.0 mm diameter; Figure 2G). A cerclage was positioned around the femoral shaft to additionally secure the chamber wings in position (Figure 2H), and the surgical wound was closed in three layers.

#### Explantation of Chambers and Processing of Constructs

Ten days (groups D/D irrad) or 12 weeks (other groups) after CSFD surgery, the acceptor animals were deeply anesthetized, and the vascular system was perfused with a radiopaque silicon mixture (Microfil^®^ Yellow, MV-122; Flow Tech Inc., Carver, MA, USA) as described previously [12]. A lateromedial and anteroposterior radiography was performed for each animal to evaluate correct positioning of the chamber and signs of osseous infection (Supplementary Figure S3). The femur/construct specimen was excised from the surrounding soft tissue, the titanium chamber was carefully opened and removed from the underlying tissue, and the peripheral edges of the construct were dissected and snap-frozen in liquid nitrogen for later RNA isolation. The remaining specimen (femur and construct) was fixed in 4% formaldehyde solution for 24 h, followed by decalcification in 20% EDTA solution (Carl Roth GmbH, Karlsruhe, Germany) in an ultrasonic device (Sonocool 255, Bandelin, Berlin, Germany).

### 2.7. RNA Isolation and Gene Expression Analysis

RNA was isolated from the construct edges of constructs in groups C and C irrad and further processed as described previously [12]. Phenol (Trizol reagent, Life Technologies, Darmstadt, Germany) and chloroform (Th. Geyer GmbH, Renningen, Germany) were used to extract RNA after homogenization in a vibrating cup mill. RNA was precipitated with isopropanol (Th. Geyer GmbH), washed with ethanol and purified using a commercially available kit, RNase free DNase Set and RNeasy Mini Kit; both Qiagen, Venlo, Netherlands). RNA integrity was confirmed on a non-denaturing agarose gel, and 1 µg of RNA per sample was used for reverse transcription (Revert Aid First-Strand cDNA Synthesis Kit Thermo Fisher Scientific, Schwerte, Germany). The expression of the following markers was determined: osteogenic markers: osteocalcin (OC), osteopontin (OPN), alkaline phosphatase (ALP), collagen I (Coll I); vascularization markers: vascular endothelial growth factor A (VEGF-A), hypoxia-inducible factor 1 alpha (Hif1a); inflammatory marker: tumor necrosis factor-alpha (TNFa); fibrosis marker: transforming growth factor-beta 1 (TGFb1). SYBR green (SSO advanced, Biorad Laboratories, Munich, Germany) and a light cycler device (C1000 Touch, CFX96 Real-Time System; Bio-Rad Laboratories) were used for quantification, and GAPDH (glyceraldehyde 3-phosphate dehydrogenase) and PPIA (peptidylprolylisomerase A (cyclophilin A)) were used as reference genes. Gene expression was normalized to a mean of 14 healthy, unirradiated bone tissue samples from a previous study [12]. Primer sequences: please see Supplementary Table S2.

### 2.8. Microcomputertomography

Two decalcified specimens per group were subjected to a microCT evaluation as described previously [12]. Microfil^®^-filled vessels were detected by a high-resolution X-ray tungsten tube operating at 80 kV and 100 mA without additional filtering (Skyscan 1172; Bruker microCT, Kontich, Belgium). The voxel size was 6.78 µm. The reconstruction of the raw data was done via reconstruction software (NRecon Client 1.6.01 and Server 1.6.01; Skyscan).

### 2.9. Histological Processing

After sufficient decalcification, the specimens were bisected along the longitudinal axis of the femur through the center of the femur/construct. Three-micrometer cross-sections were obtained from this standardized cutting plane (1 mm in both directions). Standard hematoxylin and eosin staining and Masson Goldners’ trichrome staining for each specimen were performed according to standard protocols. Immunohistochemical staining for CD68, lectin and alpha-smooth muscle actin was performed as described previously (two slides per construct) [13]. Briefly, for lectin staining, a biotinylated Lectin antibody was used (Isolectin B4 Biotin labeled, Sigma Aldrich, Munich, Germany; 1:270 in TRIS buffer) and the detection of isolectin binding was done with a streptavidin AB Complex/HRP (Dako GmbH, Hamburg, Germany), followed by DAB+ chromogen (Dako GmbH, Hamburg, Germany) and counterstaining with hematoxylin solution. For CD68, a primary antibody solution was used (monoclonal mouse anti-rat CD68, Serotec, Düsseldorf, Germany; 1:300), followed by an enhancement reagent (Zytochem Plus AP Polymer Kit) and an AP-coupled secondary antibody (anti-mouse/anti-rabbit, 30 min), development with a Fast Red solution and counterstaining with hematoxylin solution. The α-smooth muscle actin (ASMA) staining was performed using an anti-mouse-α-smooth muscle actin (SMA) antibody (Clone 1A4, Zytomed Systems; 1:300), followed by second alkaline phosphatase-labeled anti-mouse antibody (AP-Polymer), Fast Red TR/Naphthol AS (Sigma) substrate and hematoxylin for counterstaining. Images from histological staining were obtained using an Olympus IX81 microscope (Olympus, Hamburg, Germany). The bone area was measured in each construct using ImageJ software (National Institutes of Health, Bethesda, MD, USA; version 1.47v) and normalized to the total construct area (expressed as “relative bone area”). The number and the total area of Microfil^®^-filled structures were counted using ImageJ using H&E stained slides (two per construct). The vessel area was normalized to the total construct area (expressed as “relative vessel area”). The average size of vessels was calculated by dividing the total Microfil^®^-filled area by the counted number of Microfil^®^-filled structures (expressed as “average vessel size”).

In specimens containing mesenchymal stem cells (DiI labeled), the staining of apoptotic cells by TdT-mediated dUTPbiotin nick end labeling (TUNEL) was performed (FragEL DNA Fragmentation Detection Kit, Fluorescent TdT Enyzme, Calbiochem/Merck-Millipore, Darmstadt, Germany). Viable nuclei were counterstained with DAPI staining solution (Sigma Aldrich). Fluorescence images were taken with a Leitz DRMBE microscope (Leica Microsystems, Wetzlar, Germany) and a Leica DFC420 camera. Red-fluorescent (DiI positive) and TUNEL positive cells were counted using ImageJ to evaluate the survival of the implanted mesenchymal stem cells.

### 2.10. Statistics

GraphPad Prism (version 5) was used for statistical comparisons. The normal distribution of data was analyzed by means of a Kolmogorov–Smirnov Test. Histological scores for the relative bone area, vessel number (= Microfil^®^-filled structures) and DiI positive cells were compared between irradiated and the according unirradiated specimens by means of an unpaired *t*-test or a Mann–Whitney U Test. ANOVA/Bonferroni’s Post Hoc or Kruskal-Wallis/Dunn’s Post Hoc were used for comparisons between irradiated groups, as well as between unirradiated groups. Relative gene expression was compared between group C and C irrad by means of an unpaired *t*-Test or a Mann–Whitney-U Test. To evaluate whether the expression significantly differed from bone tissue, a one-sample *t*-test or a Wilcoxon Signed Rank Test was used (bone tissue set 1). A *p*-value ≤ 0.05 was considered significant for all statistical comparisons.

## 3. Results

### 3.1. Evaluation of the Animal Model

All donor animals tolerated the subcutaneous implantation of the titanium chamber without complications. A small piece of the chamber’s edge without skin coverage had developed in six animals at the time of explantation, probably due to pressure atrophy of the covering skin tissue. However, no signs of infection were present in these animals.

After CSFD surgery, 74 of 77 acceptor animals (96%) survived until the expected date of explantation. One unirradiated animal had developed severe respiratory distress two days after the CSFD surgery, and two irradiated animals were euthanized during CSFD surgery due to breakage of the femur while handling. Infection was present in four animals at explantation (5.4%; all unirradiated animals). A hypertrophic proximal femoral stump was noted in four cases (5.4%; three unirradiated and one irradiated animal), whereas instability of the femur/construct axis was present in seven cases (9.9%; all irradiated animals).

**Figure 3:**
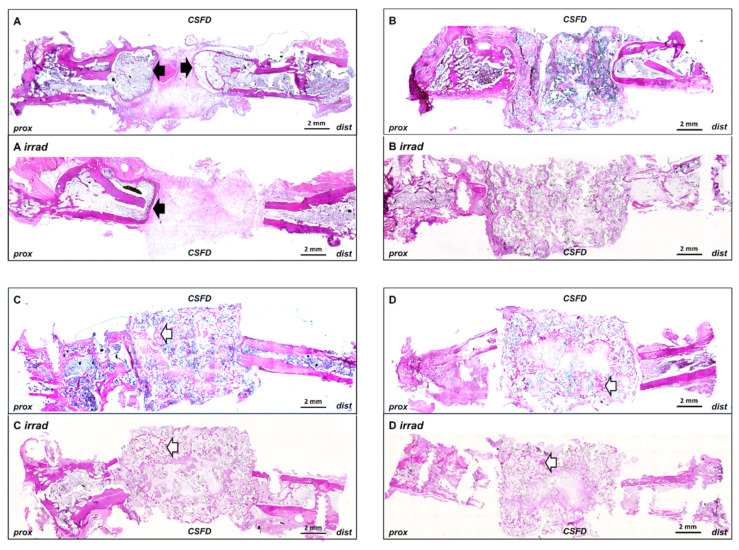
Bone formation in implanted constructs. The newly formed bone tissue was clearly visible in group C/C irrad and D/D irrad (white arrows), whereas no bone formation was induced by the scaffold alone (B/B irrad). Outgrowth of bone tissue from the femoral stumps was present in group A but very limited in group A irrad (black arrows). ((**A**–**C**): 12 weeks after CSFD, (**D**): 10 days after CSFD). Scale bar 2 mm. Merged images. Total magnification ×40.

**Figure 4:**
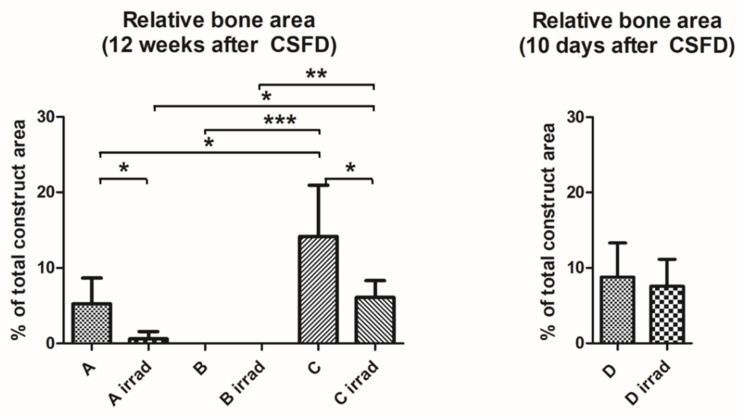
Relative bone area in constructs after 12 weeks (group A/A irrad, B/B irrad, C/C irrad) and 10 days (group D/D irrad). Bone formation was significantly impaired in group A irrad, compared to group A (* *p* ≤ 0.05). No bone formation was noted in constructs filled with hydroxyapatite scaffold alone (B/B irrad). Compared to the short-term groups (D/D irrad), the relative bone area increased in unirradiated defects (group C), whereas cessation of ossification occurred in irradiated bones (C irrad). This led to a significantly higher relative bone area in group C compared to group C irrad (* *p* ≤ 0.05). The implantation of hydroxyapatite/BMP-2/MSCs led to a significantly higher ossification than in empty defects or defects filled with hydroxyapatite alone, both with and without irradiation (* *p* ≤ 0.05; ** *p* ≤ 0.01, *** *p* ≤ 0.001). Values are given as mean ± SD.

As expected, the applied ionizing radiation led to severely impaired bone healing. In group A irrad, only very limited outgrowth of bone from the femoral stump into the empty defect was noted (Figure 3A, black arrows), whereas in group A, clear bone growth towards the gap was seen. The difference in bone growth between group A and group A irrad (5.27 ± 3.40% vs. 0.61 ± 0.98% of total defect area) was statistically significant (Figure 4). No spontaneous bridging of the femoral defect was observed after 12 weeks (group A/A irrad), proving that the defect was critically sized.

**Figure 5:**
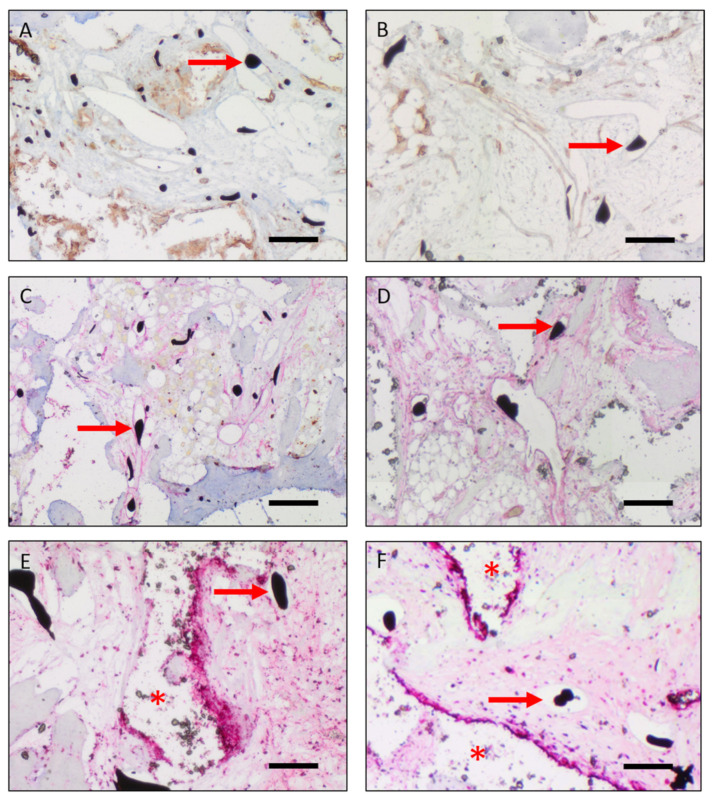
Representative immunohistochemical staining of constructs of group C (**A**,**C**,**E**) and C irrad (**B**,**D**,**F**). ((**A**,**B**): Lectin staining; (**C**,**D**): alpha-smooth muscle actin staining; (**E**,**F**): CD68 staining). (**A**–**D**): Vessel lumina filled with Microfil^®^ (black dots, red arrows) were lectin and alpha-smooth muscle actin positive in groups C and C irrad. (**E**,**F**): In groups C and C irrad, there were CD68 positive cells around the granule detectable. (Arrows: Microfil^®^ inside the vessel lumen; Asterisk: degraded scaffold). Scale bar 0.2 mm. Total magnification ×40.

Qualitative analysis of CD68 staining showed CD68 positive cells at the junction area between the construct and the femoral stumps in the 10-day groups and around the granule and screws in the 12-week groups. Overall there were more CD68 positive cells in the 10-day groups than in the 12-week groups, showing early inflammation after 10 days and resolving inflammation after 12 weeks (Figure 5E,F). In addition, the irradiated constructs showed a decreased CD68 staining compared to the non-irradiated animals.

Analgesia was adequate in all animals, both after subcutaneous implantation and CSFD surgery, as the animals showed no signs of discomfort and moved freely after surgery. However, a transient and mild loss of weight was observed after CSFD surgery.

### 3.2. Bone Formation

**Figure 6:**
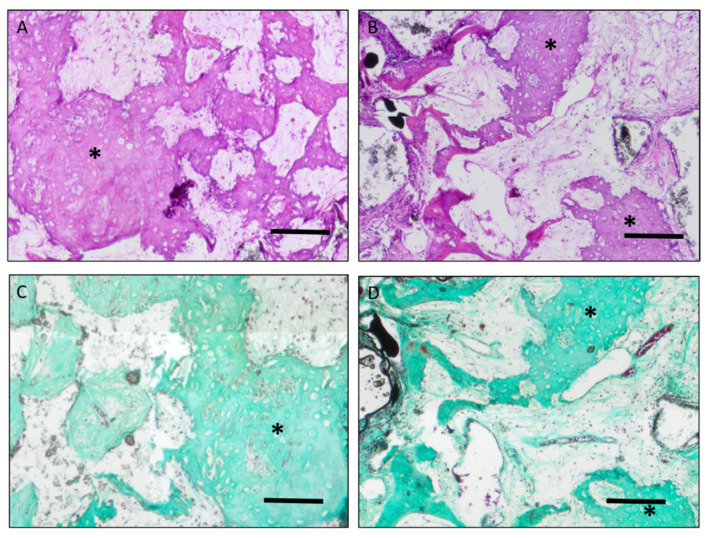
H&E and Masson Goldners’ trichrome staining of bone formations in group C and C irrad (Asterisk: bone formations; (**A**,**C**): group C; (**B**,**D**): group C irrad, (**A**,**B**): H&E staining; (**C**,**D**): Masson Goldners’ trichrome staining). Scale bar 0.2 mm. Total magnification ×40.

**Figure 7:**
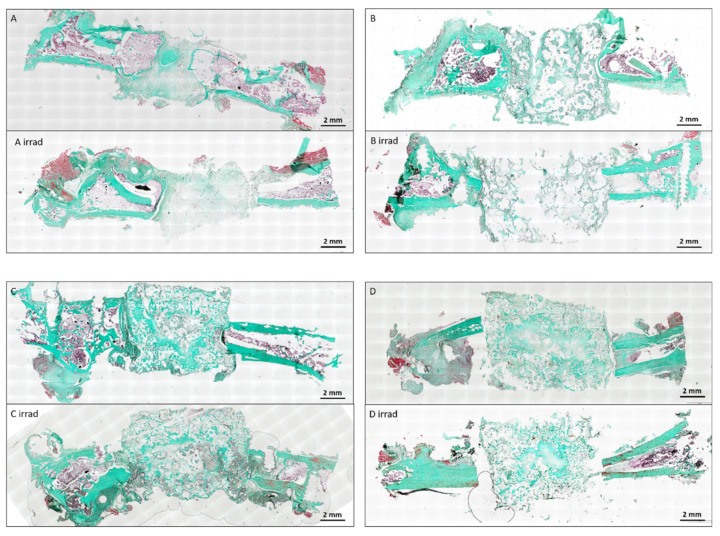
Masson Goldners’ trichrome staining of bone formations. The bone generation was detectable in group C/C irrad and D/D irrad, whereas no bone formation was induced by the scaffold alone (B/B irrad). Outgrowth of bone tissue from the femoral stumps was present in group A but very limited in group A irrad (black arrows). ((**A**–**C**): 12 weeks after CSFD, (**D**): 10 days after CSFD). Scale bar 2 mm. Merged images. Total magnification ×40.

Bone formation was evaluated by automated measurement of bone tissue in histological images and normalized to the construct area (Figure 3, Figure 6 and Figure 7).

Acceptor animals of group D and D irrad were euthanized 10 days after CSFD surgery to evaluate the amount of preossification of the implanted constructs in donor animals. Bone tissue was clearly visible in all constructs, originating from the periphery of the construct (Figure 3, arrows), demonstrating that the combination of bone-marrow-derived MSCs, BMP-2 and hydroxyapatite scaffolds resulted in significant preossification. A relative bone area of 8.8 ± 4.5% was present in group D, compared to 7.6 ± 3.6% in group D irrad (Figure 4). The difference between these two groups was not statistically significant. Twelve weeks after CSFD surgery, bone tissue was clearly visible in the groups containing hydroxyapatite scaffold/BMP-2/MSCs (group C/C irrad; Figure 3, white arrows; Figure 6, asterisks). No ossification was noted in constructs containing only the hydroxyapatite scaffold (group B/B irrad), regardless of previous irradiation (Figure 3B). Furthermore, bony outgrowths were detected in group A predominantly at the proximal stump. In quantitative evaluation, the amount of bone tissue had further increased in unirradiated defects (group C), whereas cessation of ossification was noted in group C irrad (Figure 4). The relative bone area was significantly higher in group C (14.16 ± 6.77%) than in group C irrad (6.09 ± 2.21%). The combination of a hydroxyapatite scaffold, BMP-2 and MSCs resulted in a significantly higher bone formation both in irradiated and unirradiated defects (C/C irrad), compared with both empty defects (A/A irrad) and hydroxyapatite filled chambers alone (B/B irrad; Figure 4).

### 3.3. Vascularization

**Figure 8:**
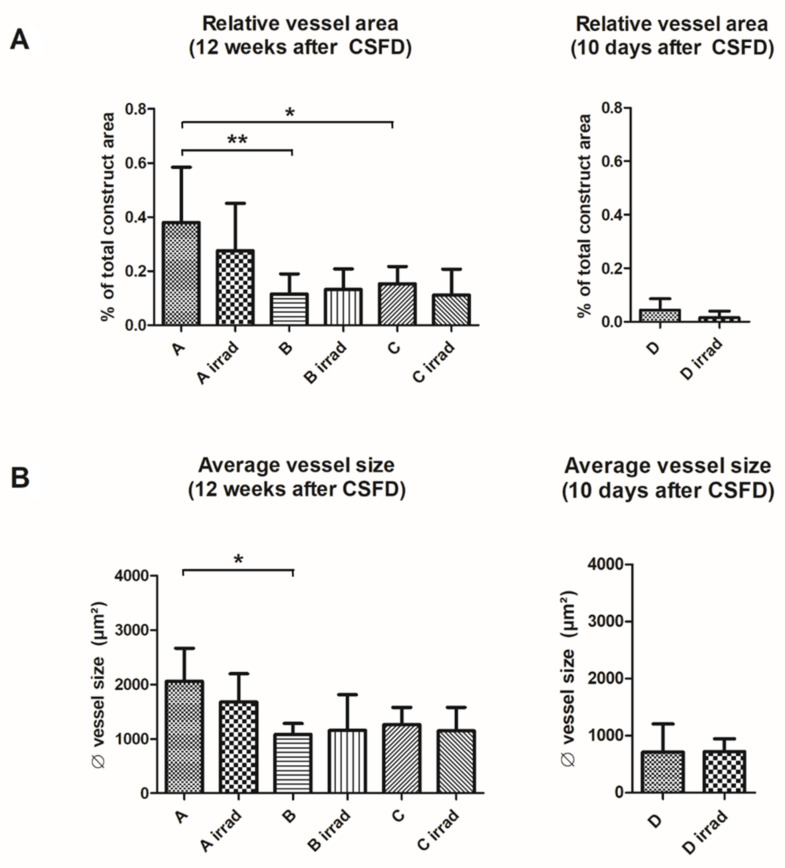
Vascularization of implanted constructs 12 weeks (group A/B/C, A irrad/B irrad/C irrad) and 10 days (D/D irrad) after CSFD surgery. (**A**) The relative vessel area was low in short-term groups D and D irrad and increased after 12 weeks in all groups. Empty defects (group A) showed a significantly higher relative vessel area than defects filled with hydroxyapatite alone (group B; ** *p* ≤ 0.01) or hydroxyapatite/BMP-2/MSCs (group C; * *p* ≤ 0.05). The differences between irradiated defects and unirradiated controls were not significant. (**B**) Similar to the relative vessel area, vessels in empty defects (group A) were significantly larger than hydroxyapatite-filled defects (group B; * *p* ≤ 0.05). The differences between irradiated and unirradiated groups were not significant. Values are given as mean ± SD.

Vascularization was evaluated by counting and measuring vascular structures filled with Microfil^®^ in H&E stained histological slides. Further, vessels were detected using lectin and alpha-smooth muscle actin stainings (Figure 5A–D). Ten days after CSFD surgery, a low relative vessel area (= total Microfil^®^ filled area per construct area) was noted both with and without irradiation (Figure 8A). The relative vessel area was smaller in irradiated defects; however, the difference was not statistically significant (*p* = 0.07). The average vessel size did not differ between group D and D irrad (Figure 8B).

After 12 weeks, a significantly higher relative vessel area was present in group A due to an ingrowth of fibrovascular tissue from the periphery and the femoral stumps, compared to groups B and C (Figure 8A). This trend was similar, although not statistically significant, in irradiated groups (*p* = 0.07). Group B and C, as well as B irrad and C irrad, did not differ regarding their relative vessel area. The average vessel size after 12 weeks (Figure 8B) was higher in group A than in group B. The difference between group C and A/B, as well as differences between irradiated groups, were not statistically significant.

The visualization of vascular structures in the µCT scan showed the ingrowth of vessels into the constructs (please see Supplementary Figure S4). However, hydroxyapatite scaffold remnants were visible on microCT scans after decalcification and interfered with the quantitative evaluation of the scanned vascular structures. Therefore, no quantification of the structures was done.

### 3.4. Cell Survival

**Figure 9:**
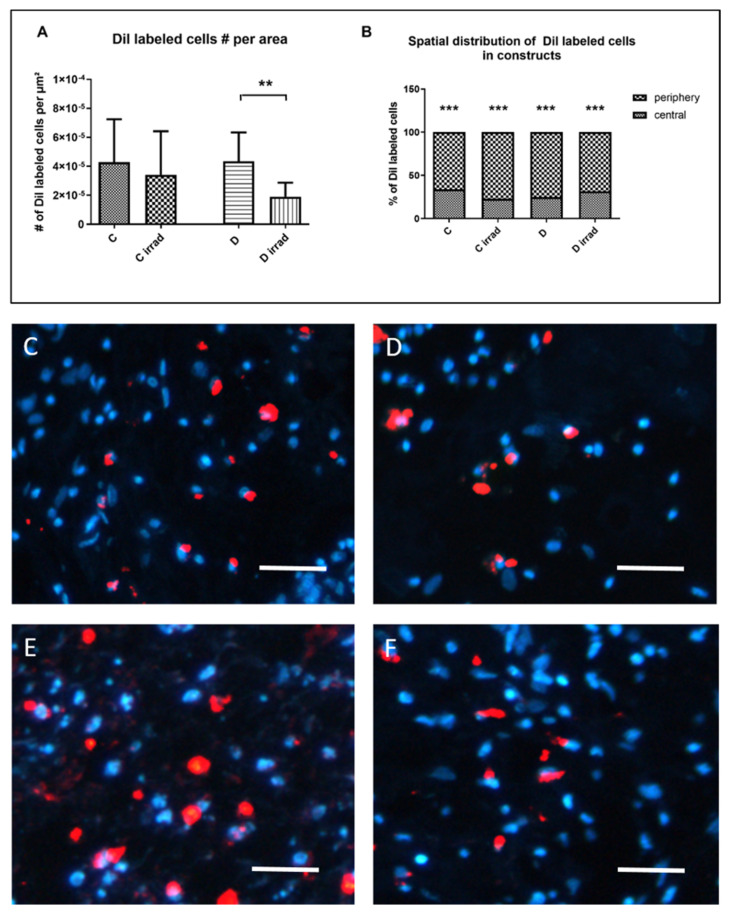
Survival of implanted, DiI labeled osteogenic MSCs in constructs, 12 weeks (group C/C irrad) and 10 days (D/D irrad) after CSFD surgery. (**A**) A significantly lower cell survival was noted in irradiated femora 10 days after CSFD, compared to unirradiated controls (** *p* ≤ 0.01). After 12 weeks, the difference was not significant. (**B**) In all groups, a significantly higher number of cells survived in the periphery compared to the center of constructs (*** *p* ≤ 0.001). However, the spatial distribution did not differ between different groups. Values are given as mean ± SD. (**C**–**F**): Representative images of DiI positive cells (red) and DAPI staining (blue) ((**C**): group C; (**D**): group C irrad, (**E**): group D; (**F**): group D irrad) (Total magnification ×100, Scale bar 50 µm).

DiI labeled cells were clearly visible in fluorescence microscopy both 10 days and 12 weeks after CSFD surgery. When normalized to the total construct area (Figure 9A), a significantly lower cell survival in irradiated defects was noted 10 days after surgery (D/D irrad), whereas the difference was not significant after 12 weeks (C/C irrad). The spatial distribution of implanted MSCs in the construct (Figure 9B) was similar in all groups, with 23% to 34% of cells located in the center of the construct and 66% to 77% located in the peripheral region. The number of cells in the periphery was significantly higher than in the center in all groups. In total the DiI positive cell density (cells/µm^2^) after 12 weeks was 4.28 × 10^−5^ in group C and 3.42 × 10^−5^ in group C irrad, whereas after 10 days, the cell density was 4.36 × 10^−5^ in group D and 1.90 × 10^−5^ in group D irrad. In the TUNEL staining, only a few cells were positive, with no differences among the different groups (data not shown).

### 3.5. Gene Expression Analysis

**Figure 10:**
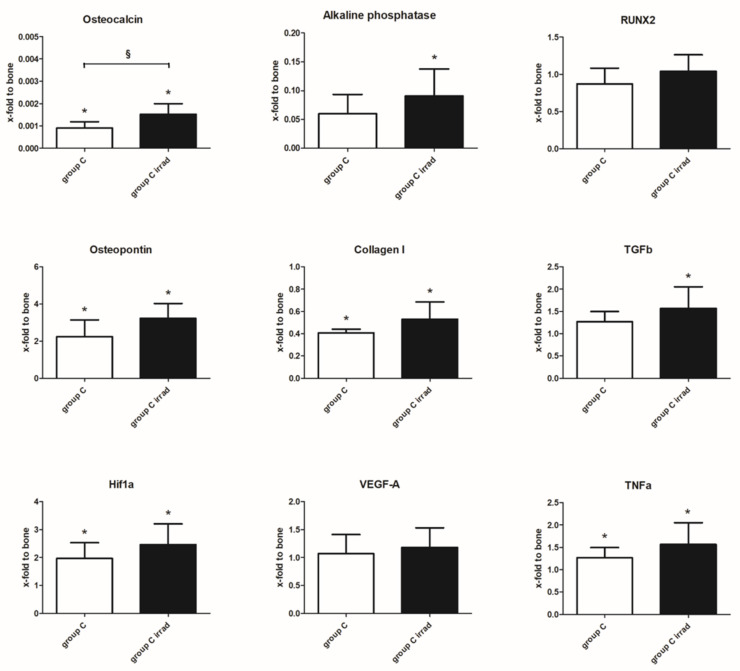
Gene expression of osteogenic, fibrotic, hypoxic, angiogenic and inflammatory markers in irradiated and unirradiated constructs, compared to healthy bone tissue (bone tissue set 1). A significant up or downregulation in comparison to bone tissue is marked with * (*p* ≤ 0.05). A significant difference in osteocalcin expression between group C and C irrad was noted (§, *p* ≤ 0.05). Values are given as mean ± SD.

Tissue specimens were taken from group C and C irrad, and the expression of various osteogenic, angiogenic, fibrotic and inflammatory markers was compared to healthy bone tissue on an mRNA level (Figure 10).

The expression of osteogenic markers, osteocalcin and alkaline phosphatase, was significantly lower in constructs than in bone tissue, whereas osteopontin expression was significantly upregulated. Analysis of the transcription factor RUNX2 did not show differences between constructs and bone tissue. Collagen I, the main type of collagen in bone, was significantly lower in both groups than in bone tissue. The fibrotic marker TGFb was slightly upregulated compared to bone; this effect, however, was significant only in group C irrad. Hif1a, an important marker for hypoxia, was significantly higher than in healthy bone tissue, whereas angiogenic marker VEGF-A was unchanged. The inflammatory marker TNFa was higher in constructs than in bone tissue, which only reached significance in group C irrad.

When comparing group C to group C irrad, the implantation of preossified constructs into irradiated defects resulted in gene expressions similar to unirradiated controls. A significantly higher expression of osteocalcin was present in group C irrad. An overall trend of an increased expression of osteogenic, fibrotic, inflammatory and hypoxic markers was noted in irradiated defects, which, however, was not statistically significant.

## 4. Discussion

### 4.1. Animal Model

The current study describes the use of a novel model of critical size femoral defects in irradiated rat hindlimbs. The critical size of the femoral defect has been confirmed in previous studies by our group, and the irradiation procedure has been established by comparing different protocols [11,12]. We found out that a single irradiation with 20 Gy had an impact on the osteogenesis and angiogenesis after 4 weeks of femoral bones compared to repetitive irradiations of 3 × 10 Gy; therefore, we used a single irradiation with 20 Gy 4 weeks prior to transplantation in this study. Overall, a good clinical outcome has been achieved by combining these two models. A very low rate of severe postoperative complications was observed, with only one animal showing severe respiratory distress that needed euthanasia. Two irradiated animals were euthanized due to breakage of the femur during surgery. This is likely due to brittle bone tissue after irradiation; thus very careful handling of the bone is necessary for irradiated animals. Due to skin atrophy over the chamber’s edge, small skin defects are possible after long-term implantation, but small defects did not necessarily lead to infection of the implant. Clinically apparent infection was more common in unirradiated femora. Irradiation can cause both local and systemic effects on the host’s immune system [14], and a lower immune reaction might have been associated to irradiation. Instability of the femur/construct axis was present in seven irradiated animals, while hypertrophy of the femoral stump was noted mainly in unirradiated controls. This underlines the decreased healing properties of irradiated bone, possibly leading to the instability of implanted constructs. However, the custom-made titanium chamber was highly suitable to bridge the defect even with limited stability, as animals were not lame prior to explantation.

### 4.2. Gene Expression Analysis

Gene expression was analyzed for group C and C irrad after isolation of RNA from peripheral areas of the construct. Attempts to isolate RNA from other groups were not successful. Due to the longer implantation period, which likely resulted in higher cellularity in the construct, it is not surprising that group C/C irrad yielded higher RNA amounts than group D/D irrad. In group B/B irrad, a lower cellularity was likely caused by the low amount of newly formed bone tissue.

We noticed an overall trend of increased gene expression in group C irrad compared to group C. The difference was statistically significant for osteocalcin. This result was similar to our previous study [12], where a general trend towards an increased gene expression was noted 8 weeks after irradiation, especially for ALP, OC, OPN and Hif1α. GAPDH and PPIA were used as reference genes and had been carefully selected based on the literature to minimize the risk for irradiation-induced regulation of our housekeeping genes [15,16]. However, regulation of the housekeeping genes cannot be fully ruled out. A recent study showed an increase in osteocyte senescence after irradiation [17]. Although PPIA has been identified as a very stable and GAPDH as fairly stable reference genes during in vitro senescence in mesenchymal stem cells [18], it is noteworthy that GAPDH expression decreases during in vivo aging in fibroblasts [19]. As in vivo molecular mechanisms of irradiation-induced osteocyte senescence have not been fully elucidated yet, we strongly suggest interpreting possible differences between group C and C irrad carefully. We also know from previous experiments that in unirradiated constructs, the gene expression of osteogenic genes decreases between the sixth and twelfth week after implantation, whereas the bone generation still increases. This might also explain the decreased gene expression levels in group C compared to group C irrad [20].

### 4.3. Bone Formation

In bone tissue engineering, a tremendous number of scaffolds have been investigated in the past years. For the present study, a commercially available, granular silicon substituted hydroxyapatite matrix (SiHA) was used. SiHA has been described as a promising scaffold for bone defect healing and for spinal fusion surgery [21,22]. However, based on the results of our current study, induction of ectopic bone formation in subcutaneous tissue by SiHA alone (group B/B irrad) seems to be rather poor. HA-based scaffolds are mainly bioactive and osteoconductive [23,24], and although osteoinductive properties of SiHA have been described [25], a limited osteoinductive potential might account for the low formation of bone tissue in the model of subcutaneous implantation without bony contact. We, therefore, need to question the use of SiHA for ectopic preossification, and we suggest implantation of such scaffolds directly into bony defects, where their osteoconductive potential might outweigh the limited osteoinduction.

The combination of 0.8% SiHA scaffold, BMP-2 and MSCs resulted in a significant bone formation both in irradiated and unirradiated defects (C/C irrad). This suggests that the SiHA-based granular matrix is a suitable scaffold for subcutaneous (ectopic) preossification if used together with osteogenic cells and growth factors. Furthermore, this is in accordance with our previous study investigating a similar scaffold (unsubstituted HA/TCP) together with MSCs/BMP-2 during ectopic preossification [20]. This study focused on the effect of irradiation on bone formations; therefore, no isolated MSC or BMP2 groups were tested. In the current study, bone formation with MSCs/BMP-2 exceeded both endogenous bone healing, expressed as ingrowth of bone tissue into empty defects (group A/A irrad) and defects filled with SiHA alone. Given the results from groups C and D, it is evident that bone formation took place not only in donor animals but also proceeded in unirradiated acceptors after transplantation. It is very likely that complete bony bridging of the critical size defect would have occurred with a longer healing time. Thus, we conclude that this combination is readily applicable for its use, especially in unirradiated tissue.

In group C irrad, on the other hand, the relative bone area was similar to group D irrad, suggesting that bone formation had ceased after implantation into irradiated acceptor tissue. Bone loss and impaired bone healing are common findings after irradiation [26]. Furthermore, bone marrow tissue as an important source of regenerative cells is often infiltrated with adipose tissue, an effect we have previously described in our irradiation model as well [12]. Bone loss and bone marrow adiposity are crucial points that need to be addressed for complete healing of the critical size bone defect. Therapeutic agents, such as bisphosphonates and osteoanabolic drugs have been used to overcome this challenge. Bisphosphonates are able to increase bone density, to improve microarchitecture and to reduce apoptosis of osteoblasts and osteocytes [27,28]. Osteoanabolic agents, such as parathyroid hormone and sclerostin antibody, are able to decrease bone marrow adipose tissue, to stimulate trabecular bone formation and to exert positive effects on osteoblasts [29,30,31]. The administration of these drugs in combination with the implantation of a SiHA/MSCs/BMP-2 construct could potentially ameliorate the outcome in irradiated acceptor animals and might be investigated in future studies.

Although silicon substituted hydroxyapatite has been described to induce the expression of alkaline phosphatase and osteocalcin in osteoblasts [32], we found only a very low expression compared to healthy bone tissue in groups C and C irrad. Similarly, collagen type I was downregulated, although to a lesser extent than ALP and OC. Conversely, osteopontin expression was upregulated in constructs, maybe due to increased inflammation. Moreover, this might indicate that a rather immature osteoblast was present in our constructs, as osteopontin is a marker of early osteoblast differentiation, while collagen type I, alkaline phosphatase and osteocalcin are expressed during later stages of osteoblast maturation [33]. With ongoing healing, OC/ALP/Collagen I expression might have further increased. Nevertheless, pronounced bone formation was present in both groups, leading us to the assumption that the expressed levels were sufficient for osteoinduction.

### 4.4. Vascularization and Cell Survival

Vascularization of tissue-engineered constructs is crucial for both survival of implanted cells and bone formation. In previous studies, the quantification of vessel number and size was performed in microCT reconstructions [12]. In the specimens of this study, however, remnants of SiHA scaffold were present after decalcification and impeded three-dimensional reconstruction. Longer decalcification times would have led to a decrease in the quality of histological staining. Therefore, we limited the evaluation of vascularization to the automated counting of vessels on histology slides.

After ten days, the total vessel area was higher in group D, compared to group D irrad, while the average vessel size was similar between these groups. The decrease in total vessel area clearly shows impaired vascularization. A lack of angiogenesis vascularization is a known issue in irradiated tissue, and especially small vessels seem to be radiosensitive [34]. In our previous study, we noticed a decrease of small-sized vessels (<20 µm diameter) after irradiation [12]. As we evaluated vascularization of constructs by counting in histological images only, we might not have been able to detect changes in such small vessels in the present study, which might explain the similar average size between groups. A central blood supply, such as an arteriovenous loop, might overcome limited vascularization, as it has been shown that 83% of blood vessels connect to the central blood vessel within a few weeks and can thus be kept patent during construct transfer into the defect [35]. A central vascular supply should significantly improve cell survival in the center of the construct [11], as seen especially in impaired tissue in group D irrad. However, a good overall cell survival was achieved in the present model, and viable cells per area did not further decrease between group D and C (D irrad and C irrad, respectively), thus surviving for a total of 18 weeks in vivo. In groups A and A irrad there was an ingrowth of fibrovascular tissue from the periphery and the femoral stumps, leading to an increased vessel density compared to the other groups also due to the missing matrix components.

The expression of Hif1a was increased in both irradiated and unirradiated specimens, compared to healthy bone tissue. This is not surprising, given the presumed hypoxic environment of the implanted tissue-engineered construct during ongoing vascularization. Taken into account that RNA was isolated from the periphery, with a comparably higher blood supply, it might have been even more pronounced in the center of the construct. Notably, the VEGF expression was not increased in our specimens. An increase in VEGF expression has been shown under hypoxic conditions [36,37], and mesenchymal stem cells are known to secrete VEGF, especially when in contact with endothelial cells [38]. We, therefore, expected a significant release of VEGF from implanted cells. However, the secretion of VEGF by MSCs is not only dependent on hypoxia. First, the degree of osteogenic differentiation of MSCs is of importance, with an increase in VEGF expression during osteogenic differentiation [37]. Based on ALP and OC expression, implanted cells in the current study were in a rather early stage of osteoblast differentiation, and VEGF might have increased with ongoing differentiation. Furthermore, the senescence of MSCs might play an important role, as the ability to secrete VEGF decreases with increasing passage numbers [39]. To overcome MSC senescence and to increase the release of angiogenic factors, in vitro treatment with substance P has been recently described and might be an interesting option for the current model as well [39]. At last, VEGF secretion can be improved in future studies by co-implantation of endothelial cells or endothelial progenitor cells, which might, in turn, be beneficial for osteogenesis [40,41,42].

### 4.5. Inflammation

The inflammatory marker TNFα was increased in implanted constructs, compared to healthy bone tissue. TNFα is known to attract muscle-derived stromal cells, which possess a high osteogenic potential and support healing in healthy and osteoporotic bone tissue [43,44]. Thus, a moderate secretion might be beneficial. However, a tight balance is needed, as TNFα is inhibitory at higher concentrations [43,45]. Ideally, early inflammation with TNFα secretion would be followed by the resolution of the inflammatory state [46]. Further, CD 68 staining showed increased CD68 positive cell numbers in the 10-day groups compared to the 12-week groups, also indicating early inflammation. The latter can be promoted by the application of pre-conditioned (licensed) mesenchymal stem cells or by anti-inflammatory cytokines, such as interleukin 4 or interleukin 13 [46]. However, the exact timing is a matter of ongoing research, and further studies are needed to allow for immunomodulation in the present model.

## 5. Conclusions

In the present study, we successfully combined a critical-sized femoral defect model with an irradiation protocol in rats, which will be of high value in tissue engineering research. Crucial points, such as impaired bone healing, limited vascularization and immunomodulation, can be investigated in the current model. The combination of SiHA, MSCs and BMP-2 resulted in the significant ectopic formation of bone tissue, which can easily be transferred into the femoral defect by means of a titanium chamber.

## Figures and Tables

**Table 1:** Study design.

Group	Prevascularization/Preossification in Donors	Chamber Content	# of Donor Animals	Irradiation of Acceptors	Vasc/oss in CSFD	# of Acceptor Animals
A	No	empty	0	no	12 weeks	7
A-irr	No	empty	0	yes (4 weeks before CSFD)	12 weeks	10
B	yes (6 weeks)	Sc/Fib	11	no	12 weeks	11
B-irr	yes (6 weeks)	Sc/Fib	10	yes (4 weeks before CSFD)	12 weeks	10
C	yes (6 weeks)	Sc/Fib/BMP/MSC	9	no	12 weeks	10
C-irr	yes (6 weeks)	Sc/Fib/BMP/MSC	10	yes (4 weeks before CSFD)	12 weeks	10
D	yes (6 weeks)	Sc/Fib/BMP/MSC	10	no	10 days	9
D-irr	yes (6 weeks)	Sc/Fib/BMP/MSC	10	yes (4 weeks before CSFD)	10 days	10

Sc = scaffold (silicon substituted hydroxyapatite), Fib = fibrinogen, BMP = recombinant bone morphogenetic protein 2, MSC = mesenchymal stem cells after early osteogenic differentiation.

## Data Availability

The data presented in this study are available on request from the corresponding author.

## Supplementary Materials

The following are available online at https://www.mdpi.com/article/10.3390/cells10092256/s1. Figure S1: ALP Staining of MSCs. Figure S2: Chamber design to enable complete removal of femoral stumps and construct (arrows indicating construct removal). Figure S3: Lateromedial (left) and anteroposterior (right) radiography of the CSFD after construct implantation, showing the correct positioning of the chamber, screws and cerclage wire. T = tibia, P = pelvis. Figure S4: Three-dimensional reconstruction of the Microfil®-filled vascular network in constructs after µCT scan. Ingrowing vessels were clearly visible (yellow). However, a quantitative evaluation was impaired by radiopaque hydroxyapatite remnants (black). Table S1: Antibodies used for flow cytometry of bone-marrow-derived mesenchymal stem cells. Table S2: Primer sequences.

## Appendix A

### Appendix A.1. Supplemental Information: Dosages of Medication for Acceptor and Donor Animals

#### Appendix A.1.1. Implantation of Titanium Chamber for Preossification/Prevascularization

Donor animals were anesthetized by inhalation of isoflurane (Forene, Abbott, Wiesbaden, Germany) in pure oxygen. After induction of anesthesia, meloxicam (0.2 mg s.c.; Metacam, Boehringer-Ingelheim, Ingelheim, Germany), tramadol hydrochloride (4 mg s.c.; Tramal, Grünenthal, Aachen, Germany) and penicillin/dihydrostreptomycin (0.2 mL s.c., Veracin compositum, Albrecht, Aulendorf, Germany) were administered for analgesia and prevention of infection. Anesthesia was maintained by isoflurane, supported by a single administration of xylazine (1.4–1.6 mg i.m.; Rompun 2%, Bayer, Leverkusen, Germany). During all surgical procedures, animals were placed on a heating plate and monitored by pulse oximetry.

Postoperative analgesia was achieved by oral tramadol hydrochloride (75 mg per liter of drinking water) for 10 days. No postoperative antibiotic treatment was deemed necessary.

#### Appendix A.1.2. Irradiation of Acceptor Animals

Acceptor animals were anesthetized by an intramuscular injection of 9 mg ketamine (Ketavet, Pfizer, Karlsruhe, Germany) and 1.8 mg xylazine (Rompun, Bayer, Leverkusen, Germany), followed by subcutaneous injection of 2 mg medetomidine (Domitor, Pfizer, Karlsruhe, Germany) to prolong anesthesia time. After irradiation, the animals were allowed to recover on a heat plate and kept in single cages for the rest of the experiment. No medication was necessary after irradiation, as animals only showed mild skin erythema without signs of pain after the procedure.

#### Appendix A.1.3. CSFD Surgery

Acceptor animals were anesthetized by use of isoflurane in pure oxygen as described above and received clindamycin (10 mg s.c.; Clindamycin, Hameln Pharma, Hameln, Germany) for the prevention of infection and a combination of buprenorphine (12 µg s.c.; Temgesic, RB Pharmaceuticals, Berkshire, UK), meloxicam (0.2 mg s.c.; Metacam, Boehringer, Ingelheim, Germany) and xylazine (1.6 mg; Rompun, Bayer, Leverkusen, Germany) for analgesia.

Postoperative analgesia was achieved using buprenorphine (18 µg BID s.c.) and meloxicam (0.2 mg SID s.c.) for 3 days, followed by tramadol hydrochloride via drinking water (75 mg per liter) for an additional 2 weeks. Prevention of infection was achieved by clindamycin (10 mg BID s.c.) for three days, followed by enrofloxacin via drinking water (Baytril, Bayer, Leverkusen, Germany; 100 mg per liter) for an additional 5 days.
