# Supplementary figures and images for: Free Transplantation of a Tissue Engineered Bone Graft into an Irradiated, Critical-Size Femoral Defect in Rats
Source: Cells. 2021 Aug 31;10(9):2256. doi: 10.3390/cells10092256 (PMC8467400; doi:10.3390/cells10092256)

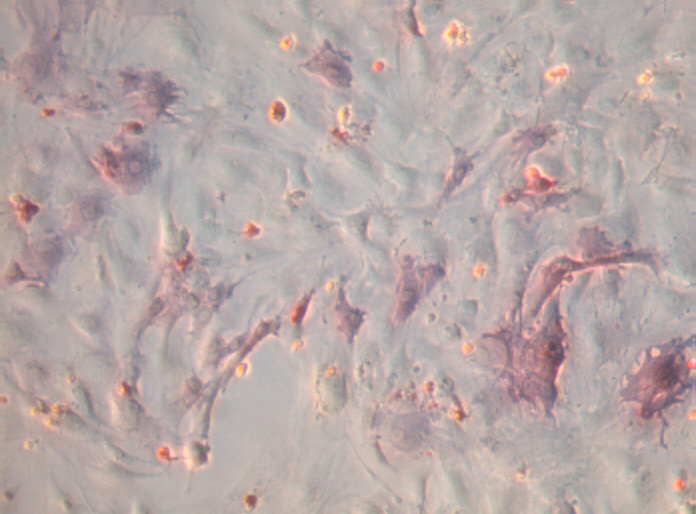

Supplement: Supplementary file 1 [file cells-10-02256-s001.zip › Supplementary Figure S1 ALP staining MSC.jpg]

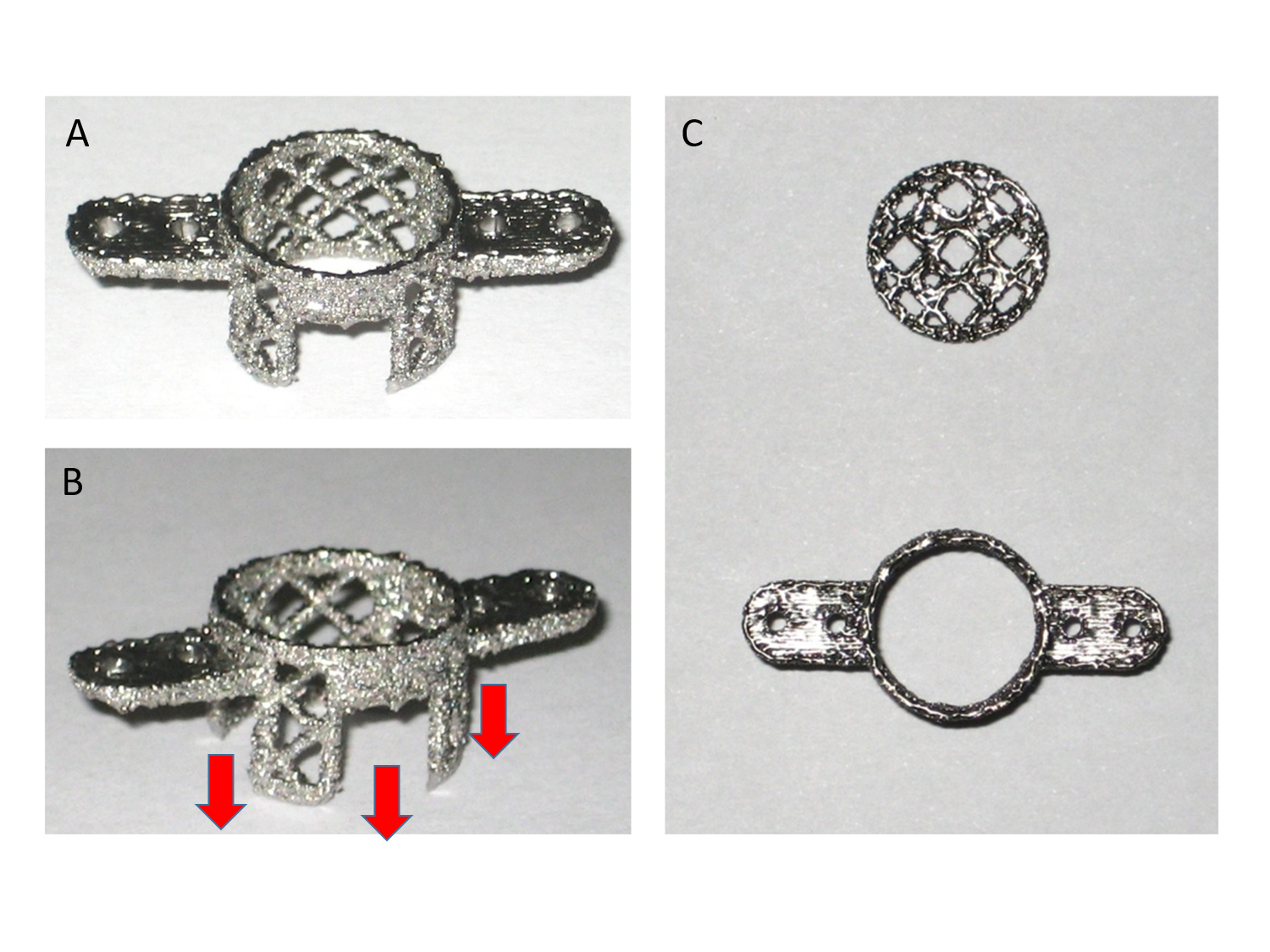

Supplement: Supplementary file 1 [file cells-10-02256-s001.zip › Supplementary Figure S2.tif]

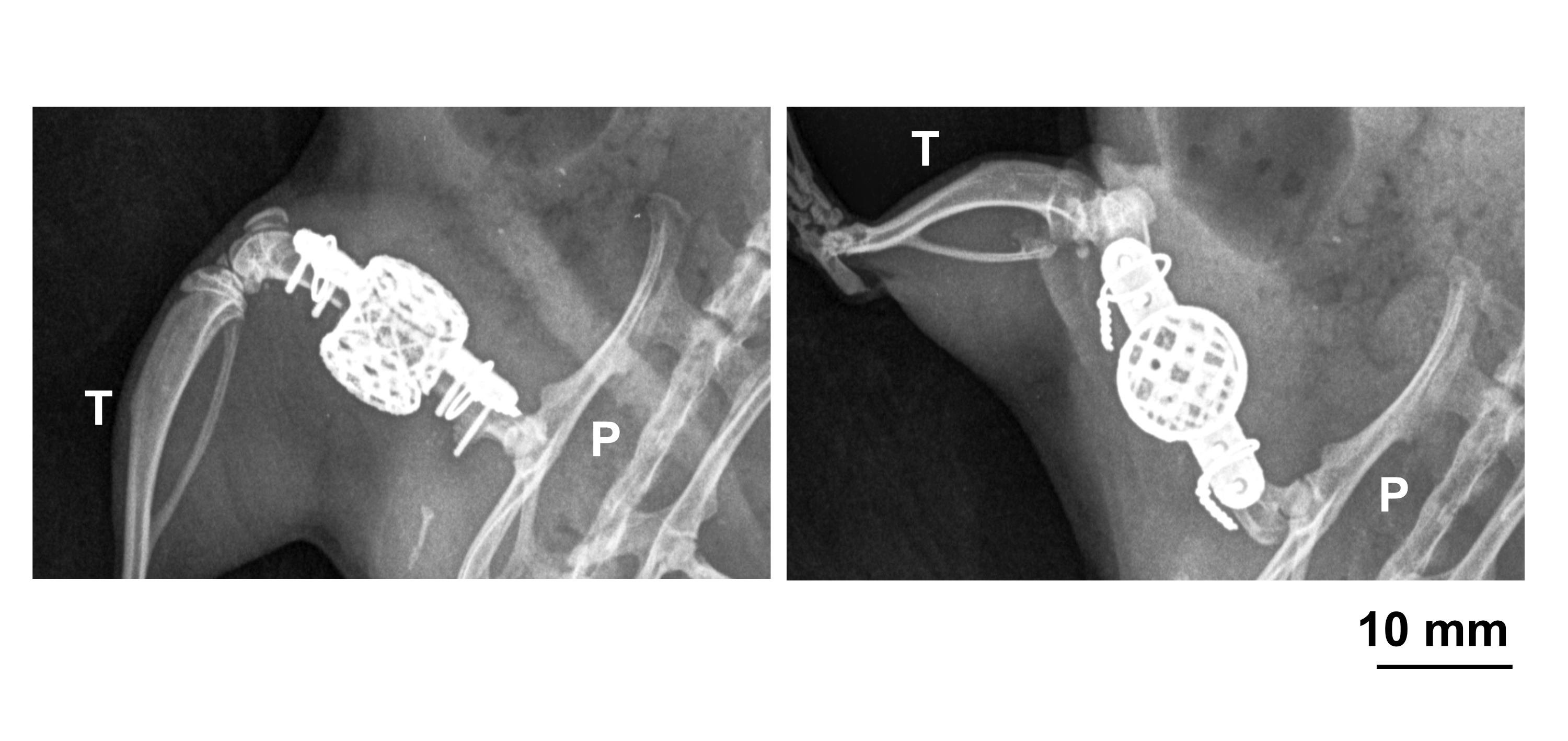

Supplement: Supplementary file 1 [file cells-10-02256-s001.zip › Supplementary Figure S3.tif]

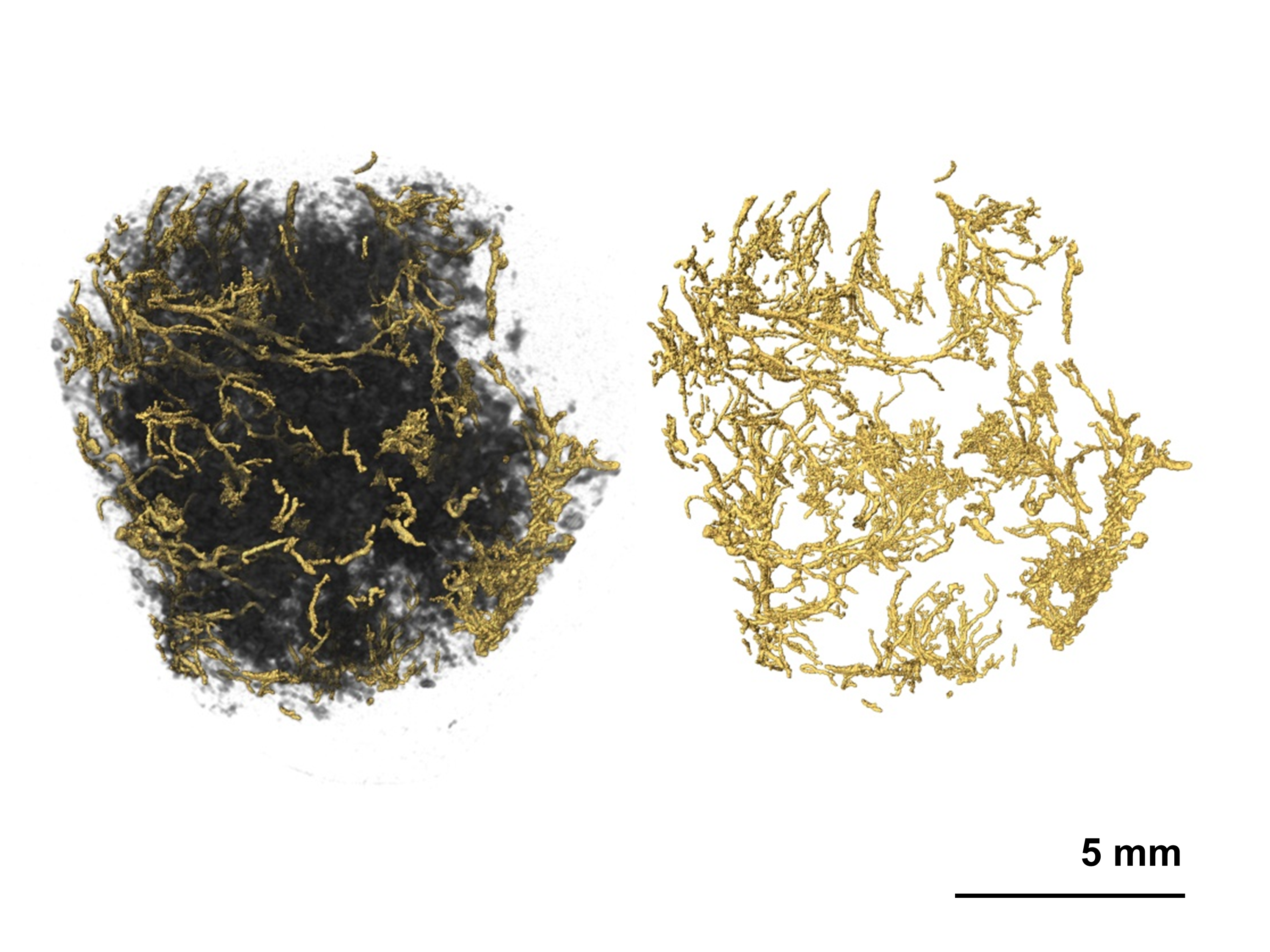

Supplement: Supplementary file 1 [file cells-10-02256-s001.zip › Supplementary Figure S4.tif]

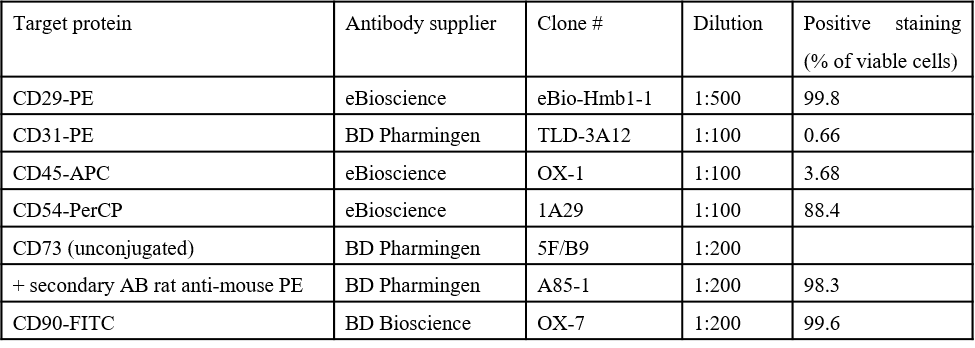

Supplement: Supplementary file 1 [file cells-10-02256-s001.zip › Supplementary Table S1.tif]

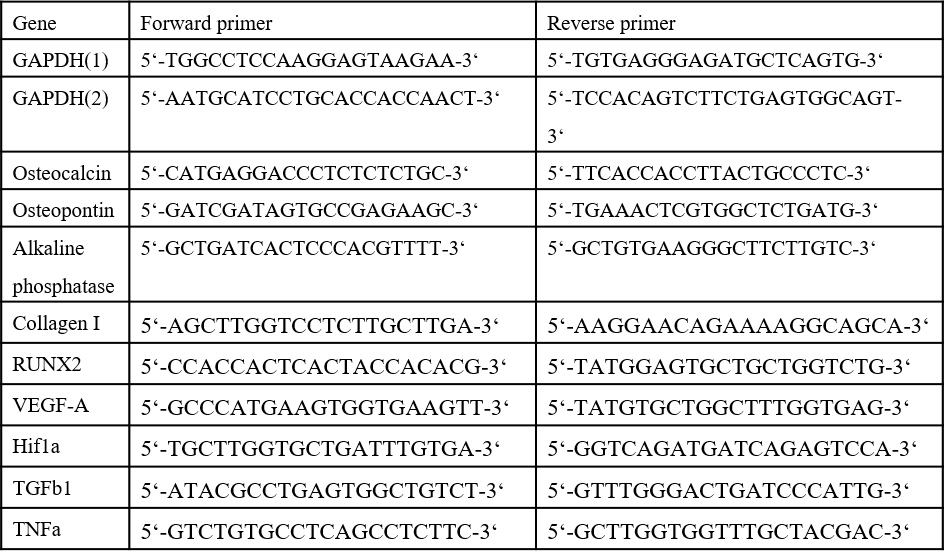

Supplement: Supplementary file 1 [file cells-10-02256-s001.zip › Supplementary Table S2.tif]
